# Midterm outcome and strength assessment after quadriceps tendon refixation with suture anchors

**DOI:** 10.1007/s00590-022-03218-x

**Published:** 2022-02-09

**Authors:** Stephanie Geyer, Felix Winden, Alexander Braunsperger, Florian Kreuzpointner, Benjamin D. Kleim, Sebastian Lappen, Andreas B. Imhoff, Julian Mehl, Maximilian Hinz

**Affiliations:** 1 Department of Orthopaedic Sports Medicine, Klinikum Rechts der Isar, Technical University of Munich, Ismaninger Straße 22, 81675 Munich, Germany; 2 Prevention Center, Department of Sport and Health Sciences, Technical University of Munich, Munich, Germany

**Keywords:** Knee injury, Sports injury, Isometric strength measurement, Acute, Chronic, Surgical treatment

## Abstract

**Purpose:**

Quadriceps tendon ruptures (QTR) occur predominantly in middle-aged patients through violent eccentric contraction that occurs either when trying to regain balance or during a fall on the hyperflexed knee. The aim of this study was to quantify midterm postoperative results, including strength potential measured via standardized strength tests following acute (< six weeks) quadriceps tendon refixation using suture anchors.

**Methods:**

All consecutive patients with QTR who underwent surgical suture anchor refixation between 2012 and 2019 at a single institution with a minimum follow-up of 12 months were retrospectively evaluated. Outcome measures included Tegner Activity Scale (TAS), Lysholm score, International Knee Documentation Committee subjective knee form (IKDC), Knee injury and Osteoarthritis Outcome Score (KOOS) subscales, return to work rates, and Visual Analog Scale (VAS) for pain. Additionally, a standardized clinical examination and an isometric strength assessment of knee extension and flexion were performed.

**Results:**

A total of 17 patients (median age 61.0 [25–75% IQR 50.5–72.5]) were available for final assessment at a mean follow-up of 47.1 ± SD 25.4 months. The majority of patients were male (82.4%) and most injuries occurred due to a fall on the hyperflexed knee (76.5%). The average time interval between trauma and surgery was 12.7 ± 7.5 days. Patients achieved a moderate level of activity postoperatively with a median TAS of 4 (3–5.5) and reported good to excellent outcome scores (Lysholm score: 97 (86.5–100); IKDC: 80.7 ± 13.5; KOOS subscales: pain 97.2 (93.1–100), symptoms 92.9 (82.5–100), activities of daily living 97.1 (93.4–100), sport and recreation function 80 (40–97.5) and knee-related quality of life 87.5 (62.5–100). All patients were able to fully return to work and reported little pain [VAS: 0 (0–0)]. No postoperative complications were reported. Strength measurements revealed a significant deficit of knee extension strength in comparison to the contralateral side (*p* = 0.011).

**Conclusion:**

Suture anchor refixation of acute QTR leads to good functional results and high patient satisfaction without major complications. Isometric knee extension strength, however, may not be fully restored compared to the unaffected side.

## Introduction

Quadriceps tendon ruptures (QTR) are generally rare injuries (2.8/100,000 person-years) that occur predominantly in middle-aged men [17]. Often, these injuries are linked to predisposing risk factors such as diabetes mellitus, renal failure, secondary hyperparathyroidism, and obesity [3, 18]. The foremost mechanism of injury reported in middle-aged patients is simple fall [6]. Young athletes, while infrequently affected by QTR, most often suffer injury due to an eccentric overload to the flexed knee [2]. Following QTR, immediate surgical treatment is recommended considering the debilitating nature of the injury [6]. In acute cases, available repair techniques include arthroscopic or open suture anchor refixation, [5, 14, 16, 19] and open transosseous repair techniques [3, 16, 21, 22]. Biomechanically, suture anchor refixation yields more favorable results due to less gap formation during cyclic loading [20] and higher ultimate failure loads [15]. At midterm follow-up, good to excellent functional outcomes and high return to work rates have been reported following open suture anchor refixation [5, 14, 16]. Only few studies, however, report on the strength outcome following suture anchor refixation in QTR with varying results: one study reports that 50–66% of patients show a relevant (> 20%) knee extension strength deficit compared to the contralateral side [16]; a second study reports a mean quadriceps strength deficit of 20% at follow-up [14].

The aim of this study was to quantify the midterm results after surgical refixation of acute QTR using suture anchors. In addition to the assessment of the clinical outcome using patient-reported outcome measures (PROMs), the functional results were evaluated by measuring both isometric knee flexion and extension strength as well as, the single-leg hop (SLH) for distance of the affected vs. the unaffected leg. The proposed hypothesis was that surgical refixation using suture anchors leads to high PROMs, good functional outcome, and high return to work rates without a relevant strength loss compared to the unaffected side in the majority of patients.

## Materials and methods

### Study cohort

**Fig. 1:**
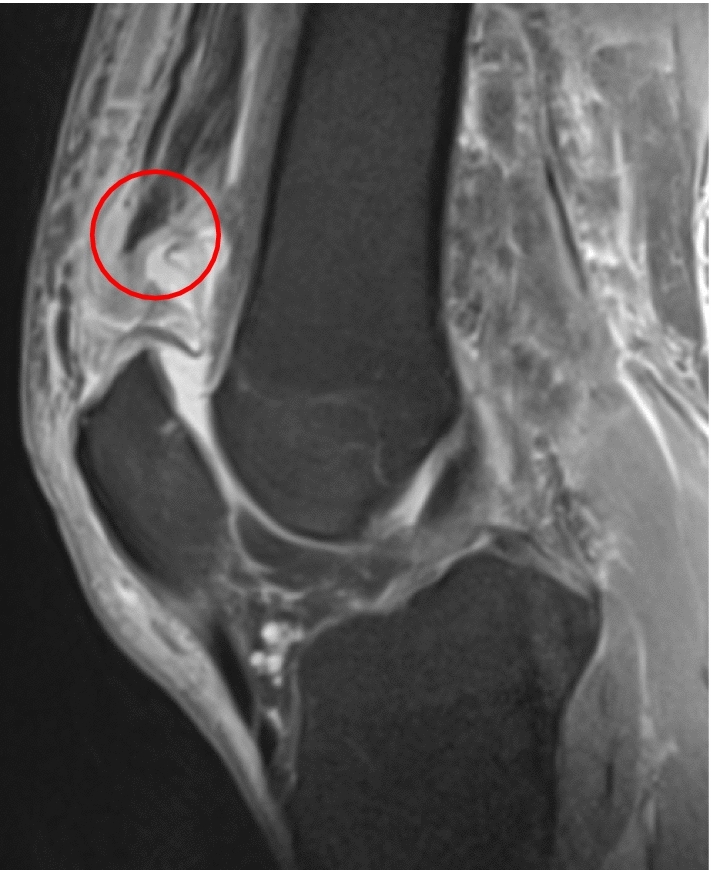
T2-weighed sagittal magnetic resonance imaging showing distal avulsion of the quadriceps tendon (red circle) (colour figure online)

Patients who underwent open surgical repair of QTR using suture anchors between September 2012 and September 2019 were included for retrospective review at a minimum follow-up of 12 months. Patients were included if an acute (< six weeks since trauma) QTR had been confirmed by both clinical examination and preoperative magnetic resonance imaging (MRI) (Fig. 1). Because of the debilitating nature of the injury, early operative intervention was performed. Exclusion criteria were chronic injuries (> six weeks since initial trauma) and recurrent QTR injuries. The study was approved by the ethics committee of the Technical University of Munich (reference: 317/20 S). It was conducted according to the Declaration of Helsinki and all patients signed informed consent forms.

## Surgical technique

All patients were operated on at a single institution by experienced sports orthopedic surgeons. Procedures were performed with the patient in a supine position.

**Fig. 2:**
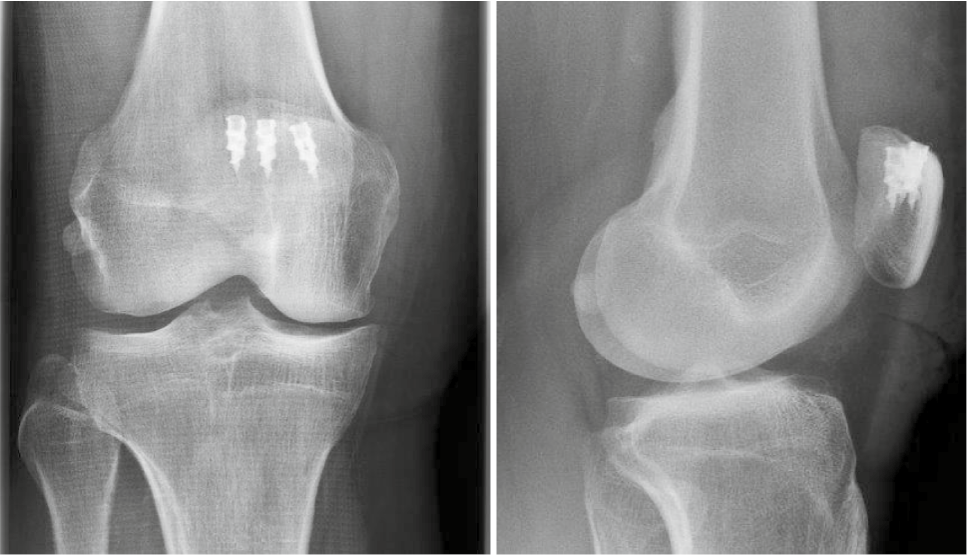
Postoperative radiographs. The antero-posterior and lateral *X*-ray of the right knee show correct anchor placement at the superior margin of the patella with three double-loaded 5.5 mm titanium suture anchors (Corkscrew^®^, Arthrex, Naples, USA)

A longitudinal skin incision was made from the superior margin of the patella in proximal direction and the fascia of the quadriceps femoris muscle was subsequently split along its fibers. The enthesis of the quadriceps muscle was visualized and scar tissue was debrided. The tendon insertion—the superior margin of the patella—was debrided and the anatomical footprint was decorticated to facilitate healing. Depending on the footprint morphology and rupture extent, two to three double-loaded 5.5 mm titanium suture anchors (Corkscrew^®^, Arthrex, Naples, USA) were used for tendon refixation (Fig. 2). The distal 5 cm of the tendon were sutured with modified Mason-Allen stitches from one strand and reduced by the pull of the second strand. The medial and lateral retinacula were refixed with the sutures of the corresponding medial and lateral suture anchor. Sutures were tied over the reduced tendon. Correct patella height was confirmed through bilateral fluoroscopy in 90° of knee flexion. Finally, the wound was irrigated and closed.

## Postoperative rehabilitation

After surgery, the affected leg was secured in an M.4 X-lock^®^ knee brace (medi GmbH & Co. KG, Bayreuth, Germany) for six weeks with knee flexion limited to 30°, 60° and 90° for 2 successive weeks, respectively. Additionally, for the first six weeks, weight bearing was restricted to ≤ 20 kg in full knee extension only. From the seventh postoperative week forward, full weight bearing was encouraged. Physiotherapy started on the first postoperative day with passive flexion to 30° (1–2 week), 60° (3–4 week), and 90° (5–6 week). Additionally, isometric quadriceps exercises in supine position with the knee in full extension were encouraged during the first six postoperative weeks. Active knee extension exercises were started from the seventh postoperative week. Patients received physiotherapy treatments 2–3 × per week.

## Outcome parameters

**Fig. 3:**
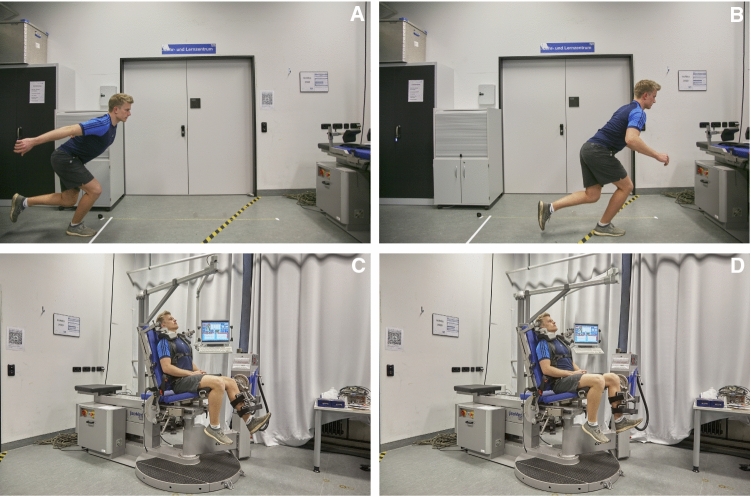
Postoperative lower extremity strength and function was evaluated by measuring the single-leg hop for distance **a** and **b**, as well as isometric knee extension **c** and knee flexion **d** strength at 60° of flexion. Images **a**, **c** and **d** were used in a prior study [10]

At follow-up, patient-reported outcome measures (PROMs), including the Lysholm score, the International Knee Documentation Committee subjective knee form (IKDC), and the Knee Injury and Osteoarthritis Outcome Score (KOOS) subscales, were collected to quantify subjective knee function. Pain was assessed using the Visual Analog Scale (VAS), and postoperative sports participation was determined by the Tegner Activity Scale (TAS). Furthermore, data on return to work rates, the level of satisfaction with the postoperative result, and information on postoperative complications, with a special focus on injury recurrence, were collected. Objectively, range of motion (ROM) of the knee, thigh circumference (10 cm and 15 cm above the knee joint line), and heel-to-buttock distance were measured to detect potential loss of muscle size or reduced flexibility. To assess lower extremity function, the SLH (Fig. 3a and b), which has been frequently used as a postoperative functional performance test following lower extremity injuries (e.g., anterior cruciate ligament tears, Achilles tendon ruptures, rectus femoris tendon ruptures, and traumatic meniscus tears [4, 7, 9–11]), was performed next. Thereafter, bilateral isometric strength was evaluated using an isokinetic dynamometer (IsoMed^®^ 2000, D&R Ferstl GmbH, Hemau, Germany). Specifically, maximum voluntary isometric contraction (MVIC), as peak torque in Newton meters (N × m), in single-joint knee extension and flexion was measured unilaterally. Subjects were seated in an upright position and secured by shoulder pads as well as hip and shoulder belts. The rotational dynamometer was calibrated to each subject’s body dimensions. The leg was positioned at the pad of the lever arm and secured with two adjustable straps. After a mock practice session using the test equipment and set-up, maximum quadriceps contraction was measured by asking the subject to extend the knee, positioned in 60° of flexion, against the measuring pad at the front of the shin for 5 sec (Fig. 2c). Maximum hamstring muscle contraction was measured by asking the subject to pull against the measuring pad in the same position for 5 seconds (Fig. 2d) [12]. MVIC_knee flexion_ and MVIC_knee extension_ were measured three times with 3- min rest intervals in between each repetition. The highest value of maximum isometric torque measured was used for data analysis, and the order in which the subject’s legs were tested was randomized [8, 12]. The hamstring to quadriceps ratio (*H*:*Q*) was determined by peak torque measures of knee flexion and extension $\left( {H:Q = \frac{{{\text{peak}}\;{\text{hamstrings}}\;{\text{torque}}}}{{{\text{peak}}\;{\text{quadriceps}}\;{\text{torque}}}} \times 100\% } \right)$ [13]. Finally, the limb symmetry index (LSI) was calculated using measurements of the injured and uninjured limb $\left( {{\text{LSI}} = \frac{{{\text{injured}}\;{\text{leg}}}}{{{\text{uninjured}}\;{\text{leg}}}} \times {100}} \right)$ [1].

## Statistical analysis

Data were analyzed using SPSS 26.0 (IBM-SPSS, New York, USA). Categorical variables are presented in sums and percentages. Normal distribution of the collected continuous variables was assessed by the Shapiro–Wilk test and graphically confirmed. Accordingly, continuous variables are presented either as mean and standard deviation (SD) or as median and 25–75% interquartile range (IQR). For group comparison of continuous variables, the Wilcoxon-test or paired *t* test was applied, depending on whether or not the data were normally distributed. Statistical significance was set at a *p* value of < 0.05.

## Results

### Demographics

Of 21 eligible patients, 17 (81.0%) were available for retrospective analysis. Three patients did not consent to participate in this study, and one patient was lost to follow-up and therefore excluded. The cohort comprised 14 male patients (82.4%) with an overall median age of 61.0 (50.5–72.5) years at index surgery. All injuries occurred either during twisting motions (23.5%) or a fall on the hyperflexed knee (76.5%). Besides being overweight, with a mean body mass index (BMI) of 29.0 ± 4.6 kg/m^2^, further predisposing factors for QTR were identified in five patients (29.4%). Four patients were former smokers and one patient suffered from type 2 diabetes mellitus. The mean time from trauma to surgery was 12.7 ± 7.5 days. The mean follow-up was 47.1 ± 25.4 months. The patients' demographics are shown in Table 1.

**Table 1:** Patient demographics

	Number of patients
Number of patients (*N*)	17
Sex (male/female)	14/3 (82% male)
BMI (kg/m^2^)	29.0 ± 4.6
Injured side (right side/left side)	10/7 (59% right side)
Age at time of surgery (years)	61.0 (50.5–72.5)
Time from trauma to surgery (days)	12.7 ± 7.5
Follow-up (months)	47.1 ± 25.4

Normally distributed continuous variables are shown as mean ± standard deviation. Non-normally distributed continuous variables are shown as median (25–75% interquartile range). Categorical variables are shown as percentages

### Patient-reported outcome measures

At follow-up, the PROMs were good to excellent (Table 2). The majority of patients were very satisfied (*N* = 9; 52.9%) or satisfied (*N* = 6; 35.3%) with the postoperative result. Two patients (11.8%) reported being very dissatisfied due to ongoing pain. Return to work was achieved by every patient. No postoperative complications or revision surgeries were reported.

**Table 2:** Patient-reported outcome measures (PROMs)

Patient-reported outcome measures	Results
VAS for pain	0 (0–0)
Lysholm score	97 (86.5–100)
TAS	4 (3–5.5)
IKDC	80.7 ± 13.5
KOOS	
Pain	97.2 (93.1–100)
Symptoms	92.9 (82.5–100)
Activites of daily living	97.1 (93.4–100)
Sport and recreation function	80.0 (40–97.5)
Knee-related quality of life	87.5 (62.5–100)

Normally distributed continuous variables are shown as mean ± standard deviation. Non-normally distributed continuous variables are shown as median (25–75% interquartile range)

*IKDC* International Knee Documentation Committee subjective knee form, *KOOS* Knee injury and Osteoarthritis Outcome Score, *TAS* Tegner Activity Scale, *VAS* Visual Analog Scale

### Functional outcome

Knee ROM, heel-to-buttock distance, thigh circumference, SLH distance, and MVIC_knee flexion_ were similar between the operated leg and the contralateral leg (*p* > 0.05). MVIC_knee extension_, however, was significantly compromised in the affected leg (*p* = 0.011). Specifically, a MVIC_knee extension_ deficit > 20% was observed in six patients (35.3%). Consequently, LSI was lower for knee extension than it was for knee flexion. *H*:*Q* showed no significant difference between legs (Table 3).

**Table 3:** Results of the strength and functional assessment

	Operated leg	Contralateral leg	*p* value
ROM knee flexion (degrees)	117.9 ± 15.8	122.5 ± 15.7	0.197
ROM knee extension (degrees)	+ 2.5 (0–6.3)	+ 5.0 (0–6.3)	1.000
Heel-to-buttock distance (cm)	24.5 ± 11.4	20.2 ± 9.4	0.161
Thigh circumference (cm)	45.9 ± 5.6	45.8 ± 5.0	0.906
SLH distance (cm)	69.7 ± 38.5	74.6 ± 41.3	0.447
MVIC_knee extension_ (N × m)	136.6 ± 73.1	179.7 ± 80.5	0.011
MCIV_knee flexion_ (N × m)	75.4 ± 24.1	82.7 ± 24.4	0.098
*H*:*Q* (%)	66.1 ± 32.0	49.8 ± 11.9	0.073
LSI			
Knee extension	79.0 ± 32.1		
Knee flexion	91.9 ± 19.7		

Normally distributed continuous variables are shown as mean ± standard deviation. Non-normally distributed continuous variables are shown as median (25–75% interquartile range)

*H:Q hamstring to quad ratio, LSI* limb symmetry index, *MVIC* maximum voluntary isometric contraction, *ROM* range of motion, *SLH* single-leg hop

## Discussion

The most important finding of this study was that patients achieved high PROMs and good functional results following suture anchor refixation of acute QTR without reported complications. Additionally, the SLH for distance, as a test for lower extremity function, did not reveal any significant deficit of the affected compared to the unaffected side. Despite these good results, however, isometric knee extension strength was not fully restored.

The subjects included in the study, with an 82.4% male gender distribution, median age at injury of 61.0 years, and generally overweight (mean BMI of 29.0 kg/m^2^), represent a typical cohort suffering from QTR [6, 16]. Furthermore, risk factors such as diabetes mellitus and smoking were identified in the present cohort in 29.4% of patients, which is consistent with previous studies (reporting between 11 and 36%) [3, 5, 16]. Mille et al. and Ristić et al. [14, 18], however, reported 80% and 85% with predisposing risk factors in their studies. This discrepancy may be partly explained by the different conditions included as risk factors between studies.

With regard to PROMs and the low rate of complication, the present study showed comparable results to several previous studies reporting on suture anchor repair of QTR [5, 14, 16]. Brossard et al. [5] reported on a series of 22 patients (25 knees) with a mean follow-up of 7 years. Patients achieved a mean Lysholm score of 92 and no cases of rerupture occurred. One patient, however, underwent revision surgery due to a painful anchor. Mille et al. [14] evaluated 11 patients prospectively for a mean timeframe of 14.7 months. At final follow-up, the majority of patients (92%) were very satisfied or satisfied with the result with a mean Lysholm score of 89.7. In total, 2 reruptures occurred, which Mille et al. tied to incompliance with wearing the prescribed splint. Furthermore, two thromboembolism-related complications occurred (1 pulmonary embolism and 1 deep vein thrombosis) [14]. Regarding transosseous repair, Boudissa et al. [3] reported comparable results in a series of 50 knees with a mean Lysholm score of 93.7, a median TAS of 4, and a 97% return to work rate (mean follow-up: 76 months), which were also consistent with our results following suture anchor repair (median Lysholm score 97, median TAS 4, 100% return to work rate, mean follow-up 47.1 months). A study by Plesser et al. [16] compared results following suture anchor repair to that following transosseous repair of QTR. Of the followed-up patients, 8 were treated with suture anchor refixation and 9 with transosseous repair. There was a tendency for lower PROMs, however, without statistical significance, in patients with suture anchor refixation (mean Lysholm 88, median TAS 4, mean IKDC 76.0 and mean VAS for pain 5) compared to patients with transosseous repair (mean Lysholm score 94, median TAS 5, IKDC 85.1, mean VAS 0) [16].

Regarding strength assessments, several studies reported on outcomes after suture anchor refixation and/or transosseous repair in patients suffering from QTR [14, 16, 22]. Following suture anchor refixation, Mille et al. [14] reported a mean quadriceps strength deficit of 20% compared to the contralateral side. In the present study, isometric knee extension and flexion strength was tested which showed that 35.3% of patients had a knee extension strength deficit > 20% compared to the unaffected side. Knee flexion strength, however, was not compromised significantly. West et al. [22] evaluated 20 QTR and 30 patellar tendon ruptures following transosseous repair (and augmentation with “relaxing sutures”) with isokinetic strength testing performed 12 months postoperatively in 23 patients. A mean quadriceps strength deficit of 35% at slow speed (60°/s) and 38.8% (240°/s) at high speed was observed. These strength results, however, were not reported separately between QTR and patellar tendon ruptures; thus, extrapolation may be limited [22]. Plesser et al.[16] reported on both suture anchor refixation (6 cases) and transosseous suture repairs (5 cases). Isokinetic strength testing revealed that 50% of patients treated with suture anchors and 20% of patients treated with transosseous sutures had a strength deficit > 20% at 60°/s. Furthermore, at a higher speed (240°/s), 4 out of 6 patients in the suture anchor group showed a strength deficit > 20% whereas no patients in the transosseous suture group demonstrated such a deficit [16].

There were several limitations to this study. The limitations of the present case series include the retrospective design with a limited number of patients. Additionally, no radiological evaluation such as an ultrasound, X-ray, or MRI was performed to evaluate tendon integrity, rule out anchor dislocation, and assess secondary patella baja or osteoarthritis at follow-up. Moreover, a comparative control-group was not included and a comparison between different repair techniques, i.e., suture anchor vs. transosseous repair, could not be made.

The strengths of the present study include that this was a single-center cohort-study with a homogenous patient cohort. A standardized surgical technique with only one type of suture anchor (5.5 mm titanium suture anchors [Corkscrew^®^, Arthrex, Naples, USA]) was used in all patients. After surgery, a standardized rehabilitation protocol was followed. Besides undergoing isometric strength measurements with an isokinetic dynamometer, each individual performed a functional task (i.e., the SLH for distance) at follow-up to evaluate lower extremity function. A reasonable midterm follow-up of 47.1 months was reached.

Overall, the findings of this study may contribute to a more informed discussion about the surgical treatment of acute QTR and prognosis following suture anchor refixation.

## Conclusion

Surgical refixation of acute QTR using suture anchors leads to good functional results and high patient satisfaction without major surgical complications; however, isometric knee extension strength may not be fully restored when compared to the unaffected side.
